# Reduced fronto–striatal white matter integrity in schizophrenia patients and unaffected siblings: a DTI study

**DOI:** 10.1038/npjschz.2015.1

**Published:** 2015-04-01

**Authors:** Max de Leeuw, Marc M Bohlken, René C W Mandl, René S Kahn, Matthijs Vink

**Affiliations:** 1 Department of Psychiatry, Brain Center Rudolf Magnus, University Medical Center Utrecht, Utrecht, The Netherlands

## Abstract

**Background:**

Schizophrenia is characterized by impairments in the fronto–striatal network. Underlying these impairments may be disruptions in anatomical pathways connecting frontal and striatal regions. However, the specifics of these disruptions remain unclear and whether these impairments are related to the genetic vulnerability of schizophrenia is not known.

**Methods:**

Here, we investigated fronto–striatal tract connections in 24 schizophrenia patients, 30 unaffected siblings, and 58 healthy controls using diffusion tensor imaging. Mean fractional anisotropy (FA) was calculated for tracts connecting the striatum with frontal cortex regions including the dorsolateral prefrontal cortex (DLPFC), medial orbital frontal cortex, and inferior frontal gyrus. Specifically, the striatum was divided into three subregions (caudate nucleus, putamen, and nucleus accumbens) and mean FA was computed for tracts originating from these striatal subregions.

**Results:**

We found no differences between patients, siblings, and controls in mean FA when taking the whole striatum as a seed region. However, subregion analyses showed reduced FA in the tract connecting the left nucleus accumbens and left DLPFC in both patients (*P*=0.0003) and siblings (*P*=0.0008) compared with controls.

**Conclusions:**

The result of reduced FA in the tract connecting the left nucleus accumbens and left DLPFC indicates a possible reduction of white matter integrity, commonly associated with schizophrenia. As both patients and unaffected siblings show reduced FA, this may represent a vulnerability factor for schizophrenia.

## Introduction

Underlying the clinical and cognitive symptoms in schizophrenia may be dysfunctions of the frontal lobe and the striatum.^1–5^ Indeed, functional magnetic resonance imaging (MRI) studies have demonstrated abnormal fronto–striatal activity.^1–3,6–9^ Moreover, structural MRI studies have revealed reductions in brain volume of the frontal cortex^10–12^ and striatum^11,13^ in schizophrenia patients.

In addition to functional and structural brain measurements, anatomical pathways connecting frontal and striatal regions may also be disrupted in schizophrenia. Two studies have investigated fronto–striatal white matter tracts using diffusion tensor imaging (DTI). Quan *et al.*^14^ reported reduced fractional anisotropy (FA) in the tract connecting the left inferior frontal gyrus (IFG) with the striatum in 16 schizophrenia patients compared with 18 matched controls.^14^ However, they only compared tracts connecting frontal regions with the whole striatum, using the striatum as a single seed region, rather than subdividing it into specific subregions. This subdivision is important as different striatal components are involved in specific functional networks.^15^ Indeed, Bracht *et al.*^16^ investigated white matter tracts connecting the nucleus accumbens with frontal and subcortical regions in schizophrenia patients (*n*=24) and controls (*n*=22).^16^ They reported on increased probability indices forming part of a bundle of interest for the tract connecting the nucleus accumbens with the dorsolateral prefrontal cortex (DLPFC), suggesting reduced white matter tract integrity. However, they did not find a difference in FA in this tract. This inconsistency makes it unclear whether and how this white matter tract is impaired by schizophrenia. Furthermore, in both the studies, it remains unclear whether these fronto–striatal white matter tract dysfunctions are related to the illness itself or to a genetic vulnerability for the disorder.

Therefore, we investigated fronto–striatal tracts in a large cohort of schizophrenia patients, unaffected siblings, and matched healthy controls using DTI. Siblings do not have the illness, but share on average 50% of their genes with their ill relative.^17^ Furthermore, they have a 10-fold increased risk to develop schizophrenia.^18^ Consequently, if siblings show impairments in fronto–striatal tract connections similar to those observed in patients this would provide evidence in support for a genetic vulnerability underlying this phenotypic abnormality. Fronto–striatal tract abnormalities in siblings are anticipated given reports on functional^1,6,19–22^ as well as structural abnormalities^10,11,23^ in this network. Moreover, abnormalities in white matter integrity have already been shown in siblings in other brain regions including the fasiculus arcuatus,^24–26^ medial frontal cortex,^27^ prefrontal cortex,^28^ cingulate and angular gyri,^29^ inferior occipitofrontal fasiculus,^25^ anterior limb of the internal capsules,^26^ corpus callosum genu,^30^ cuneus,^31^ and temporal lobe.^30^

Here, we examined FA in fronto–striatal pathways using DTI in 24 schizophrenia patients, 30 unaffected siblings, and 58 healthy controls. FreeSurfer software^32^ was used to parcellate the gray matter regions used to trace the fiber bundles of interest. We subdivided the frontal cortex into three regions: DLPFC, medial orbital frontal cortex (mOFC), and IFG, all of which are consistently reported to be abnormal in schizophrenia patients and their siblings in functional^1,8,21^ as well as structural MRI studies.^10,33^ Neurons from these frontal regions project to the caudate nucleus, putamen, and nucleus accumbens separately, together forming the fronto–striatal network.^34^ Mean FA was then computed along averaged tracts starting in each of these striatal subregions directing to the frontal cortex regions.

Given reports on reduced FA in various tracts (for recent review, see Fitzsimmons *et al.*^35^), we hypothesize that FA in fronto–striatal white matter tracts will be reduced in schizophrenia patients. Furthermore, we hypothesize that if these deficits signify a genetic vulnerability, then similar deficits are also present in unaffected siblings of schizophrenia patients.

## Materials and methods

### Participants

Twenty-four schizophrenia patients, 30 unaffected siblings, and 58 healthy control subjects participated in this study. All subjects were right-handed and there were no differences between groups for age and gender (Table 1). None of the participants had any contraindications for MRI or suffered from alcohol or drug dependence, which was assessed with the Composite International Diagnostic Interview. The patients were outpatients recruited from the Department of Psychiatry at the University Medical Center Utrecht and participating in an ongoing longitudinal study.^36^ The diagnosis of schizophrenia, schizophreniform, or schizoaffective disorder in patients was assessed with the Structured Clinical Interview for DSM-IV or the Comprehensive Assessment of Symptoms and History.^37^ Symptom severity in terms of positive, negative, and general symptoms were assessed with the positive and negative syndrome scale (PANSS).^38^ All schizophrenia patients received antipsychotic medication (medication use is listed in Supplementary Information). Four siblings had a history of at least one depressive episode, as verified by the Comprehensive Assessment of Symptoms and History. None of the healthy control subjects had a history of a neurological or psychiatric diagnosis as verified by either the Mini-International Neuropsychiatric Interview^39^ or the Schedules for Clinical Assessment in Neuropsychiatry (SCAN 2.1).^40^ Healthy control subjects who had a first-degree relative suffering from a psychotic disorder were excluded. The participants received monetary compensation for participation. All gave written informed consent. The ethics committee of the University Medical Center of Utrecht approved this study.

### Diffusion tensor imaging

#### Image acquisition and preprocessing

**Figure 1:**
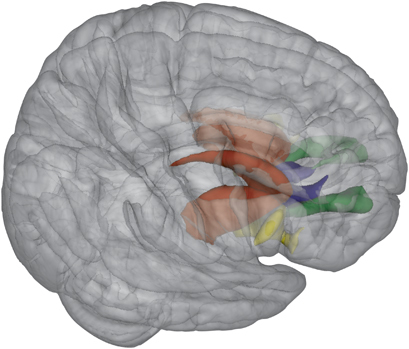
Mean fractional anisotropy was compared along averaged fibers connecting the striatum (red) with the frontal cortex regions: striatum–DLPFC (green), striatum–mOFC (blue), and striatum–IFG (yellow). Right=right.

**Figure 2:**
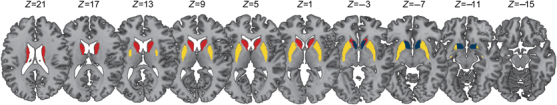
The striatum was divided into three subregions: nucleus accumbens (blue), caudate nucleus (yellow), and putamen (red), and mean fractional anisotropy was computed for tracts originating from these striatal subregions directing to frontal cortex regions including DLPFC, mOFC, and IFG. Right=right.

A T1-weighted structural MRI scan and a set of two diffusion-weighted imaging (DWI) scans were obtained from each subject using a 3.0 T Achieva scanner (Philips, Best, The Netherlands). One three-dimensional T1-weighted scan (185 slices; repetition time=8.4 ms; echo time=3.8 ms; flip angle=8°; field of view, 252×185×288 mm; voxel size: 1 mm isotropic) of the whole head was made for anatomical reference. The T1-weighted scans were used to extract anatomically delineated regions of interest (ROIs) of the caudate nucleus, putamen, nucleus accumbens, DLPFC (consisting of the rostral middle frontal gyrus^41^), mOFC, and IFG, consisting of the pars opercularis, pars orbitalis, and pars triangularis; Figure 1, Figure 2) in each hemisphere using the FreeSurfer 5.1.0 structural imaging pipeline.^32^

A set of two transverse DWI scans were acquired (30 diffusion-weighted volumes with different non-collinear diffusion directions with b-factor=1,000 s/mm^2^ and eight diffusion-unweighted volumes with b-factor=0 s/mm^2^; parallel imaging SENSE factor=2.5; flip angle=90°; 60 slices of 2.5 mm; no slice gap; 96×96 acquisition matrix; reconstruction matrix 128×128; FOV=240 mm; TE=88 ms; TR=9,822 ms; no cardiac gating; and total scan duration=296 s). The second DWI scan is identical to the first except that the k-space readout is reversed, which allows for correction of susceptibility artifacts during preprocessing. Preprocessing of the DWI scans was performed with the diffusion toolbox of Andersson *et al.*^42,43^ and in-house developed software.^44^ First, susceptibility artifacts were corrected by calculating a distortion map on the basis of the two b=0 images acquired with reversed k-space readout. Subsequently, it was applied to all DWI volumes. This resulted in one corrected DWI set consisting of a single b=0 volume (averaged over eight b=0 volumes) and 30 corrected weighted volumes.^43^ Finally the DWI set was corrected for eddy-current distortions and small head movements.^42^

#### Fronto–striatal fiber tractography and diffusion parameter reconstruction

Diffusion modeling and probabilistic tractography were carried out using the FMRIB Diffusion Toolbox (FDT, version 2.0, http://fsl.fmrib.ox.ac.uk/fsl/fsl-4.1.9/fdt/fdt_probtrackx.html). This process involves generating connectivity distributions from user-specified seed voxels to target voxels. First, the whole striatum (nucleus accumbens, putamen and caudate nucleus) was used as a seed mask and the three ROI’s of the frontal cortex (DLPFC, mOFC and IFG) were defined as target ROI’s, such that for each subject three fiber distributions from striatum to frontal cortex were obtained (Figure 1). Each frontal ROI was specified as a waypoint and as a termination mask to ensure that only those streamlines running between the seed mask and target ROI were captured in the fiber distribution. The default parameters (5000 streamline samples, step length of 0.5 mm, and curvature threshold of 0.2) were used during the probabilistic fiber tracking procedure.

Subsequently, tracts originating from the three anatomical subregions of the striatum were analyzed separately by using these predefined ROIs as separate seed masks directing to the frontal cortex regions as described above (Figure 2). In this way, a total of 12 tracts were traced for and within each hemisphere between the frontal cortex and the striatum, leaving 24 fiber distributions for each subject in total.

Because the seed points could be volumetrically dependent on individual or group differences, a group average fiber was reconstructed for each of the 24 fiber distributions. First, the Tract Based Spatial Statistics toolbox (version 1.2)^45^ was applied to subjects’ FA maps for warping into FMRIB58_FA standard space. This nonlinear registration was also applied to each of the 24 individually obtained fiber distributions. By only selecting the top 1% of streamlines in each fiber distrubution that overlapped in all participating subjects, a total of 24 group average tracts were reconstructed. The group average tracts were made binary and subsequently they were projected onto the warped FA maps, allowing for the estimation of a mean FA measure per individual per tract.

#### Statistical analysis

Demographic data between schizophrenia patients, siblings, and healthy controls were compared using independent sample *t*-tests. General linear model (GLM) analyses were performed to test for effects of group (schizophrenia patients, siblings, and controls) on FA of the tracts connecting the striatum with the frontal regions (DLPFC, mOFC, and IFG). Subsequently, similar GLMs were performed to test for effects of group (schizophrenia patients, siblings, and controls) on FA of the tracts connecting the three subregions of the striatum (caudatus, putamen, and nucleus accumbens) with the frontal regions (DLPFC, mOFC, and IFG). All the results were Bonferroni corrected for multiple testing (three seeds×three targets×two hemispheres=18), resulting a critical *P* value of 0.0028. In schizophrenia patients, Pearson’s correlations were used to calculate associations between mean FA and symptom severity, as measured with the PANSS. Finally, as it has been shown that nicotine use may impact FA measures in fronto–striatal tracts,^46^ we compared mean FA, using two-sample *t*-tests, between cigarette smokers and nonsmokers in the whole sample as well as in healthy controls, schizophrenia patients, and siblings.

## Results

### Group differences in fractional anisotropy

There were no significant differences among patients, siblings, and controls in mean FA along tracts averaged for the whole striatum with the frontal cortex regions (Table 2). However, when investigating tracts projecting from subregions of the striatum, schizophrenia patients as well as their unaffected siblings showed reduced mean FA in the tract between the left nucleus accumbens and left DLPFC (patients versus controls: *t*(80)=3.80, *P*=0.0003; siblings versus controls: *t*(86)=3.49, *P*=0.0008; Table 3). Patients and siblings did not differ (*t*(52)=0.56, *P*=0.58) indicating a similar reduction in FA. Symptoms severity in schizophrenia patients as measured with the PANNS scores did not correlate with mean FA in this tract (PANSS positive symptoms score: *r*=0.1, *P*=0.65, PANSS negative symptoms score: *r*=−0.6, *P*=0.77, PANSS general symptoms score: *r*=−0.2, *P*=0.38). Finally, smoking status did not affect mean FA for this tract in the whole sample (*t*(60)=0.68, *P*=0.50), nor in healthy controls (*t*(8)=−1.86, *P*=0.10), schizophrenia patients (*t*(20)=0.45, *P*=0.66), or siblings (*t*(28)=0.68, *P*=0.50). Mean FA in the other tracts did not differ between the groups.

## Discussion

This study investigated fronto–striatal pathways in 24 schizophrenia patients, 30 unaffected siblings, and 58 healthy controls using DTI. Results show reduced functional anisotropy (FA) in the tract connecting the left nucleus accumbens and left DLPFC in schizophrenia patients as well as their unaffected siblings, indicating reduced white matter integrity compared with controls. Taken together with the fact that siblings share on average 50% of their genes with their ill relative, these results indicate that reduced white matter integrity in this tract may represent a vulnerability factor for schizophrenia.

Our finding of reduced FA in the tract connecting the left nucleus accumbens and left DLPFC is consistent with findings from Bracht *et al.*^16^ who compared fronto–striatal tracts between 24 schizophrenia patients and 22 healthy controls. Although they did not find difference in FA, they computed a measure representing spatial extension of fiber tracts (probability indices forming part of a bundle of interest). They found this measure to be increased in schizophrenia patients compared with controls in the tract between the left nucleus accumbens and left DLPFC, and suggest this to indicate volume reduction of this white matter pathway. We replicated and extended this finding by showing decreased FA in this particular tract in schizophrenia patients. Moreover, we found a similar FA reduction in this tract in siblings of schizophrenia patients, indicating that this deficit may represent a vulnerability factor for schizophrenia.

We did not find reduced FA in schizophrenia patients or siblings when averaging over all tracts connecting the entire striatum and DLPFC. This is in line with data from Quan *et al.*,^14^ who also did not find differences between schizophrenia patients (*n*=16) and controls (*n*=18) in FA values between tracts connecting DLPFC and striatum.^14^ However, they did not investigate tracts originating from different subsections of the striatum.

Our finding of decreased mean FA in both schizophrenia patients and siblings may indicate decreased white matter integrity, as FA is used as an index for the microstructural integrity of white matter fiber bundles.^47^ White matter integrity abnormalities as measured with DTI have been reported in schizophrenia patients and their unaffected siblings in several regions of the brain including the frontal lobe, hippocampus, and internal capsule.^24–30,35^ In the present study, we extend these results by showing white matter integrity reductions in schizophrenia patients and siblings specifically in fibers connecting the left nucleus accumbens and left DLPFC. As we did not find deficits in other tracks, it is unlikely that our results are driven by a global impairment in white matter integrity. However, it should be noticed that the schizophrenia patients in our sample are relatively young, so it cannot be ruled out that progression of the illness over time may result in abnormalities of other white matter tracks.^48^

Both the nucleus accumbens and the DLPFC are part of reward pathways^49^ and are known to be involved in delayed reward processing.^50,51^ Our finding of reduced FA in this particular tract is consistent with earlier reports on impaired fronto–striatal reward processing in schizophrenia patients^8,9^ and siblings.^22,52^ Our current finding of structural impairments in the reward pathway combined with previous reports of functional fronto–striatal impairments during reward processing adds to the evidence in support of fronto–striatal deficits representing a genetic vulnerability factor for schizophrenia.

The decrease in white matter integrity in schizophrenia patients was not related with symptom severity as measured with the PANSS. This was anticipated given that Bracht *et al.* also did not find such a relationship.^16^ Furthermore, siblings showed similar white matter integrity reduction while being symptom-free. Taken together, this null finding is consistent with the notion that our finding of reduced white matter integrity in the tract connecting left nucleus accumbens and left DLPFC is related to the genetic vulnerability for schizophrenia rather than to the clinical manifestations.

Our study has several limitations which need to be addressed. Schizophrenia patients used antipsychotic medication which may have influence on white matter integrity.^53^ However, this is not expected given that we have previously failed to show an effect of medication on white matter volume in schizophrenia patients.^54^ Moreover, the fact that patients as well as unmedicated siblings show this deficit may indicate that this represents a genetic vulnerability for schizophrenia rather than a medication effect. However, common environmental factors cannot be ruled out. To quantify the influence of genetic factors on the observed reduction in white matter tract integrity, a discordant twin-design would be most suitable. As it has already been shown that white matter integrity is substantially heritable,^55^ it is likely that genetic factors have a role in the effect observed in the present study. One other potential limitation is that the patients were not acutely ill as they show moderate PANSS score. However, as unaffected siblings also show decreased FA in the tract connecting the left nucleus accumbens and left DLPFC, it is unlikely that symptom severity impacts the structural impairment we report on. In contrast, it may very well be that patients that are more severely affected by the illness display FA impairments in additional white matter tracts.

Here, we show impairments in fronto–striatal pathways in schizophrenia patients as well as in unaffected siblings. Specifically, we found mean FA to be decreased in the tract connecting the left nucleus accumbens and left DLPFC. This result is in line with the notion that schizophrenia is characterized by fronto–striatal deficits. Moreover, the present data add to the evidence suggesting that fronto–striatal deficits represent genetic vulnerability factors for schizophrenia. Future research should focus on how this network develops in adolescents at genetic risk for schizophrenia.

## Figures and Tables

**Table 1:** Demographic characteristics of the diagnostic groups

	*HC (*n*=58)*	*SB (*n*=30)*	*SZ (*n*=24)*	*Test statistic*	P
Age (years)	28.8±1.0	31.4±1.2	31.1±0.7	F=2.04	0.14
Gender (M/F)	35/23	17/13	19/5	*χ* ^2^=3.39	0.18
Participant’s education level	7.2±0.2	6.3±0.3	4.8±0.4	F=15.10	<0.001
Father’s education level	5.2±0.5	5.6±0.5	5.4±0.5	F=0.23	0.80
Mother’s education level	4.9±0.5	5.4±0.4	4.6±0.5	F=0.85	0.43
Cigarette smokers	2	13	12	F=1.68	0.20
Cigarettes per day	3.5±1.5	10.4±2.1	14.3±3.7	F=1.16	0.33
Duration of illness (years)			6.2±0.9		
Paranoid/disorganized/undifferentiated type			23/0/0		
Schizoaffective disorder			1		
Chlorpromazine equivalent doses (mg)			321.5±53.4		
PANSS positive symptoms score			14.5±0.8		
PANSS negative symptoms score			13.3±0.7		
PANSS general symptoms score			27.0±1.1		
History of depressive episode		4			

Abbreviations: F, female; HC, healthy controls; M, male; PANNS, positive and negative syndrome scale; SB, unaffected siblings of schizophrenia patients; SZ, schizophrenia patients.

Values represent mean±s.e.m. Level of education was measured on a 9-point scale ranging from no education (0) to university degree (8).

**Table 2:** Fractional anisotropy for tracts connecting the whole striatum with the frontal cortex regions

	*HC (*n*=58)*	*SB (*n*=30)*	*SZ (*n*=24)*	P *(HC versus SB)*	P *(HC versus SZ)*	P *(SB versus SZ)*
*Striatum*–*DLPFC*						
R	0.36±0.02	0.36±0.02	0.36±0.02	0.80	0.53	0.74
L	0.35±0.02	0.34±0.02	0.35±0.02	0.67	0.25	0.17
						
*Striatum*–*mOFC*						
R	0.28±0.01	0.28±0.01	0.28±0.01	0.71	0.97	0.73
L	0.26±0.01	0.26±0.01	0.26±0.01	0.61	0.87	0.53
						
*Striatum*–*IFG*						
R	0.30±0.01	0.30±0.01	0.29±0.01	0.96	0.30	0.33
L	0.28±0.01	0.28±0.01	0.28±0.01	0.47	0.93	0.49

Abbreviations: DLPFC, dorsolateral prefrontal cortex; HC, healthy controls; IFG, inferior frontal gyrus; L, left; mOFC, medial orbital frontal cortex; R, right; SB, unaffected siblings of schizophrenia patients; SZ, schizophrenia patients.

Values represent mean±s.d.

**Table 3:** Fractional anisotropy for tracts connecting subregions of the striatum with the frontal cortex regions

	*HC (*n*=58)*	*SB (*n*=30)*	*SZ (*n*=24)*	P *(HC versus SB)*	P *(HC versus SZ)*	P *(SB versus SZ)*
*Nucleus accumbens*–*DLPFC*						
R	0.38±0.02	0.37±0.02	0.37±0.02	0.16	0.22	0.92
L	0.35±0.01	0.34±0.02	0.33±0.02	0.0008*	0.0003*	0.58
						
*Nucleus accumbens*–*mOFC*						
R	0.22±0.01	0.21±0.01	0.22±0.01	0.24	0.63	0.55
L	0.21±0.01	0.21±0.02	0.21±0.01	0.82	0.80	0.72
						
*Nucleus accumbens*–*IFG*						
R	0.27±0.01	0.27±0.02	0.27±0.01	0.42	0.48	0.94
L	0.25±0.01	0.25±0.01	0.26±0.01	0.92	0.10	0.16
						
*Caudate*–*DLPFC*						
R	0.37±0.02	0.37±0.02	0.37±0.02	0.86	0.23	0.38
L	0.36±0.02	0.35±0.02	0.36±0.03	0.57	0.17	0.09
						
*Caudate*–*mOFC*						
R	0.29±0.02	0.29±0.01	0.29±0.02	0.70	0.92	0.80
L	0.28±0.02	0.28±0.01	0.29±0.02	0.92	0.48	0.55
						
*Caudate*–*IFG*						
R	0.35±0.01	0.35±0.02	0.35±0.01	0.78	0.19	0.20
L	0.33±0.01	0.33±0.02	0.33±0.01	0.72	0.95	0.72
						
*Putamen*–*DLPFC*						
R	0.39±0.02	0.39±0.02	0.39±0.02	0.89	0.75	0.86
L	0.38±0.02	0.38±0.02	0.39±0.02	0.55	0.27	0.11
						
*Putamen*–*mOFC*						
R	0.32±0.02	0.32±0.02	0.31±0.01	0.67	0.74	0.50
L	0.28±0.02	0.28±0.01	0.28±0.01	0.41	0.91	0.51
						
*Putamen*–*IFG*						
R	0.26±0.01	0.25±0.01	0.25±0.01	0.72	0.33	0.50
L	0.24±0.01	0.24±0.01	0.24±0.01	0.45	0.84	0.37

Abbreviations: DLPFC, dorsolateral prefrontal cortex; HC, healthy controls; IFG, inferior frontal gyrus; L, left; mOFC, medial orbital frontal cortex; R, right; SB, unaffected siblings of schizophrenia patients; SZ, schizophrenia patients.

P values with * survived Bonferroni correction for multiple testing. Values represent mean±s.d.

## Notes

MdL, MMB, RCWM, RSK, and MV declare no conflict of interest.

## References

[bib1] Zandbelt BB, van Buuren M, Kahn RS, Vink M. Reduced proactive inhibition in schizophrenia is related to corticostriatal dysfunction and poor working memory. Biol Psychiatry 2011; 70: 1151–1158. 21903198 10.1016/j.biopsych.2011.07.028

[bib2] Van Veelen NMJ, Vink M, Ramsey NF, Kahn RS. Left dorsolateral prefrontal cortex dysfunction in medication-naive schizophrenia. Schizophr Res 2010; 123: 22–29. 20724113 10.1016/j.schres.2010.07.004

[bib3] Van Veelen NMJ, Vink M, Ramsey NF, van Buuren M, Hoogendam JM, Kahn RS. Prefrontal lobe dysfunction predicts treatment response in medication-naive first-episode schizophrenia. Schizophr Res 2011; 129: 156–162. 21497488 10.1016/j.schres.2011.03.026

[bib4] Davis KL, Kahn RS, Ko G, Davidson M. Dopamine in schizophrenia: a review and reconceptualization. Am J Psychiatry 1991; 148: 1474–1486. 1681750 10.1176/ajp.148.11.1474

[bib5] Hoptman MJ, Antonius D, Mauro CJ, Parker EM, Javitt DC. Cortical thinning, functional connectivity, and mood-related impulsivity in schizophrenia: relationship to aggressive attitudes and behavior. Am J Psychiatry 2014; 171: 939–948. 25073506 10.1176/appi.ajp.2014.13111553 PMC4178944

[bib6] Vink M, Ramsey NF, Raemaekers M, Kahn RS. Striatal dysfunction in schizophrenia and unaffected relatives. Biol Psychiatry 2006; 60: 32–39. 16603134 10.1016/j.biopsych.2005.11.026

[bib7] Quidé Y, Morris RW, Shepherd AM, Rowland JE, Green MJ. Task-related fronto-striatal functional connectivity during working memory performance in schizophrenia. Schizophr Res 2013; 150: 468–475. 24016726 10.1016/j.schres.2013.08.009

[bib8] Nielsen MØ, Rostrup E, Wulff S, Bak N, Lublin H, Kapur S et al. Alterations of the brain reward system in antipsychotic naïve schizophrenia patients. Biol Psychiatry 2012; 71: 898–905. 22418013 10.1016/j.biopsych.2012.02.007

[bib9] Morris RW, Vercammen A, Lenroot R, Moore L, Langton JM, Short B et al. Disambiguating ventral striatum fMRI-related BOLD signal during reward prediction in schizophrenia. Mol Psychiatry 2012; 17, 235, 280–289. 10.1038/mp.2011.75 PMC3284694 21709684

[bib10] Harms MP, Wang L, Campanella C, Aldridge K, Moffitt AJ, Kuelper J et al. Structural abnormalities in gyri of the prefrontal cortex in individuals with schizophrenia and their unaffected siblings. Br J Psychiatry 2010; 196: 150–157. 20118463 10.1192/bjp.bp.109.067314 PMC2815937

[bib11] Oertel-Knöchel V, Knöchel C, Matura S, Rotarska-Jagiela A, Magerkurth J, Prvulovic D et al. Cortical-basal ganglia imbalance in schizophrenia patients and unaffected first-degree relatives. Schizophr Res 2012; 138: 120–127. 22464726 10.1016/j.schres.2012.02.029

[bib12] Buchanan RW, Vladar K, Barta PE, Pearlson GD. Structural evaluation of the prefrontal cortex in schizophrenia. Am J Psychiatry 1998; 155: 1049–1055. 9699693 10.1176/ajp.155.8.1049

[bib13] Keshavan MS, Rosenberg D, Sweeney JA, Pettegrew JW. Decreased caudate volume in neuroleptic-naive psychotic patients. Am J Psychiatry 1998; 155: 774–778. 9619149 10.1176/ajp.155.6.774

[bib14] Quan M, Lee S-H, Kubicki M, Kikinis Z, Rathi Y, Seidman LJ et al. White matter tract abnormalities between rostral middle frontal gyrus, inferior frontal gyrus and striatum in first-episode schizophrenia. Schizophr Res 2013; 145: 1–10. 23415471 10.1016/j.schres.2012.11.028 PMC4110910

[bib15] Shohamy D. Learning and motivation in the human striatum. Curr Opin Neurobiol 2011; 21: 408–414. 21658933 10.1016/j.conb.2011.05.009

[bib16] Bracht T, Horn H, Strik W, Federspiel A, Razavi N, Stegmayer K et al. White matter pathway organization of the reward system is related to positive and negative symptoms in schizophrenia. Schizophr Res 2014; 153: 136–142. 24485586 10.1016/j.schres.2014.01.015

[bib17] Meyer-Lindenberg A, Weinberger DR. Intermediate phenotypes and genetic mechanisms of psychiatric disorders. Nat Rev Neurosci 2006; 7: 818–827. 16988657 10.1038/nrn1993

[bib18] Gejman PV, Sanders AR, Kendler KS. Genetics of schizophrenia: new findings and challenges. Annu Rev Genomics Hum Genet 2011; 12: 121–144. 21639796 10.1146/annurev-genom-082410-101459

[bib19] De Leeuw M, Kahn RS, Zandbelt BB, Widschwendter CG, Vink M. Working memory and default mode network abnormalities in unaffected siblings of schizophrenia patients. Schizophr Res 2013; 150: 555–562. 24051015 10.1016/j.schres.2013.08.016

[bib20] Vink M, Zandbelt BB, Gladwin T, Hillegers M, Hoogendam JM, van den Wildenberg WPM et al. Frontostriatal activity and connectivity increase during proactive inhibition across adolescence and early adulthood. Hum Brain Mapp 2014; 35: 4415–4427. 24532023 10.1002/hbm.22483 PMC6869143

[bib21] Van Buuren M, Vink M, Rapcencu AE, Kahn RS. Exaggerated brain activation during emotion processing in unaffected siblings of patients with schizophrenia. Biol Psychiatry 2011; 70: 81–87. 21531384 10.1016/j.biopsych.2011.03.011

[bib22] de Leeuw M, Kahn RS, Vink M. Fronto-striatal dysfunction during reward processing in unaffected siblings of schizophrenia patients. Schizophr Bull 2015; 41: 94–103. 25368371 10.1093/schbul/sbu153 PMC4266310

[bib23] Mamah D, Harms MP, Wang L, Barch D, Thompson P, Kim J et al. Basal ganglia shape abnormalities in the unaffected siblings of schizophrenia patients. Biol Psychiatry 2008; 64: 111–120. 18295189 10.1016/j.biopsych.2008.01.004 PMC2486271

[bib24] Boos HBM, Mandl RCW, van Haren NEM, Cahn W, van Baal GCM, Kahn RS et al. Tract-based diffusion tensor imaging in patients with schizophrenia and their non-psychotic siblings. Eur Neuropsychopharmacol 2013; 23: 295–304. 22841128 10.1016/j.euroneuro.2012.05.015

[bib25] Kubicki M, Shenton ME, Maciejewski PK, Pelavin PE, Hawley KJ, Ballinger T et al. Decreased axial diffusivity within language connections: a possible biomarker of schizophrenia risk. Schizophr Res 2013; 148: 67–73. 23800617 10.1016/j.schres.2013.06.014 PMC3755869

[bib26] Muñoz Maniega S, Lymer GKS, Bastin ME, Marjoram D, Job DE, Moorhead TWJ et al. A diffusion tensor MRI study of white matter integrity in subjects at high genetic risk of schizophrenia. Schizophr Res 2008; 106: 132–139. 18849149 10.1016/j.schres.2008.09.016

[bib27] Camchong J, Lim KO, Sponheim SR, Macdonald AW. Frontal white matter integrity as an endophenotype for schizophrenia: diffusion tensor imaging in monozygotic twins and patients’ nonpsychotic relatives. Front Hum Neurosci 2009; 3: 35. 19893757 10.3389/neuro.09.035.2009 PMC2773169

[bib28] Hao Y, Yan Q, Liu H, Xu L, Xue Z, Song X et al. Schizophrenia patients and their healthy siblings share disruption of white matter integrity in the left prefrontal cortex and the hippocampus but not the anterior cingulate cortex. Schizophr Res 2009; 114: 128–135. 19643580 10.1016/j.schres.2009.07.001

[bib29] Hoptman MJ, Nierenberg J, Bertisch HC, Catalano D, Ardekani Ba, Branch Ca et al. A DTI study of white matter microstructure in individuals at high genetic risk for schizophrenia. Schizophr Res 2008; 106: 115–124. 18804959 10.1016/j.schres.2008.07.023

[bib30] Wang Q, Deng W, Huang C, Li M, Ma X, Wang Y et al. Abnormalities in connectivity of white matter tracts in patients with familial and non-familial schizophrenia. Psychol Med 2011; 41: 1691–1700. 21205362 10.1017/S0033291710002412

[bib31] Moran ME, Luscher ZI, McAdams H, Hsu JT, Greenstein D, Clasen L et al. Comparing fractional anisotropy in patients with childhood-onset schizophrenia, their healthy siblings, and normal volunteers through DTI. Schizophr Bull 2015; 41: 66–73. 25217482 10.1093/schbul/sbu123 PMC4266298

[bib32] Fischl B, Salat DH, van der Kouwe AJW, Makris N, Ségonne F, Quinn BT et al. Sequence-independent segmentation of magnetic resonance images. Neuroimage 2004; 23 Suppl 1: S69–S84. 15501102 10.1016/j.neuroimage.2004.07.016

[bib33] Byun MS, Kim JS, Jung WH, Jang JH, Choi J-S, Kim SN et al. Regional cortical thinning in subjects with high genetic loading for schizophrenia. Schizophr Res 2012; 141: 197–203. 22998933 10.1016/j.schres.2012.08.028

[bib34] Postuma RB, Dagher A. Basal ganglia functional connectivity based on a meta-analysis of 126 positron emission tomography and functional magnetic resonance imaging publications. Cereb Cortex 2006; 16: 1508–1521. 16373457 10.1093/cercor/bhj088

[bib35] Fitzsimmons J, Kubicki M, Shenton ME. Review of functional and anatomical brain connectivity findings in schizophrenia. Curr Opin Psychiatry 2013; 26: 172–187. 23324948 10.1097/YCO.0b013e32835d9e6a

[bib36] Genetic Risk and Outcome in Psychosis (GROUP) Investigators. Evidence that familial liability for psychosis is expressed as differential sensitivity to cannabis: an analysis of patient-sibling and sibling-control pairs. Arch Gen Psychiatry 2011; 68: 138–147. 20921112 10.1001/archgenpsychiatry.2010.132

[bib37] Andreasen NC, Flaum M, Arndt S. The Comprehensive Assessment of Symptoms and History (CASH). An instrument for assessing diagnosis and psychopathology. Arch Gen Psychiatry 1992; 49: 615–623. 1637251 10.1001/archpsyc.1992.01820080023004

[bib38] Kay SR, Fiszbein A, Opler LA. The positive and negative syndrome scale (PANSS) for schizophrenia. Schizophr Bull 1987; 13: 261–276. 3616518 10.1093/schbul/13.2.261

[bib39] Sheehan DV, Lecrubier Y, Sheehan KH, Amorim P, Janavs J, Weiller E et al. The Mini-International Neuropsychiatric Interview (M.I.N.I.): the development and validation of a structured diagnostic psychiatric interview for DSM-IV and ICD-10. J Clin Psychiatry 1998; 59 Suppl 2: 22–33, quiz 34–57. 9881538

[bib40] Wing JK, Babor T, Brugha T, Burke J, Cooper JE, Giel R et al. SCAN. Schedules for Clinical Assessment in Neuropsychiatry. Arch Gen Psychiatry 1990; 47: 589–593. 2190539 10.1001/archpsyc.1990.01810180089012

[bib41] Kikinis Z, Fallon JH, Niznikiewicz M, Nestor P, Davidson C, Bobrow L et al. Gray matter volume reduction in rostral middle frontal gyrus in patients with chronic schizophrenia. Schizophr Res 2010; 123: 153–159. 20822884 10.1016/j.schres.2010.07.027 PMC2975427

[bib42] Andersson JLR, Skare S. A model-based method for retrospective correction of geometric distortions in diffusion-weighted EPI. Neuroimage 2002; 16: 177–199. 11969328 10.1006/nimg.2001.1039

[bib43] Andersson JLR, Skare S, Ashburner J. How to correct susceptibility distortions in spin-echo echo-planar images: application to diffusion tensor imaging. Neuroimage 2003; 20: 870–888. 14568458 10.1016/S1053-8119(03)00336-7

[bib44] Mandl RCW, Schnack HG, Luigjes J, van den Heuvel MP, Cahn W, Kahn RS et al. Tract-based analysis of magnetization transfer ratio and diffusion tensor imaging of the frontal and frontotemporal connections in schizophrenia. Schizophr Bull 2010; 36: 778–787. 19042913 10.1093/schbul/sbn161 PMC2894583

[bib45] Smith SM, Jenkinson M, Johansen-Berg H, Rueckert D, Nichols TE, Mackay CE et al. Tract-based spatial statistics: voxelwise analysis of multi-subject diffusion data. Neuroimage 2006; 31: 1487–1505. 16624579 10.1016/j.neuroimage.2006.02.024

[bib46] Savjani RR, Velasquez KM, Thompson-Lake DGY, Baldwin PR, Eagleman DM, De La Garza IiR et al. Characterizing white matter changes in cigarette smokers via diffusion tensor imaging. Drug Alcohol Depend 2014; 145C: 134–142. 10.1016/j.drugalcdep.2014.10.006 25457737

[bib47] Basser PJ, Pierpaoli C. Microstructural and physiological features of tissues elucidated by quantitative-diffusion-tensor MRI. J Magn Reson B 1996; 111: 209–219. 8661285 10.1006/jmrb.1996.0086

[bib48] Kochunov P, Glahn DC, Rowland LM, Olvera RL, Winkler A, Yang Y-H et al. Testing the hypothesis of accelerated cerebral white matter aging in schizophrenia and major depression. Biol Psychiatry 2013; 73: 482–491. 23200529 10.1016/j.biopsych.2012.10.002 PMC3645491

[bib49] Sesack SR, Grace AA. Cortico-basal ganglia reward network: microcircuitry. Neuropsychopharmacology 2010; 35: 27–47. 19675534 10.1038/npp.2009.93 PMC2879005

[bib50] McClure SM, Laibson DI, Loewenstein G, Cohen JD. Separate neural systems value immediate and delayed monetary rewards. Science 2004; 306: 503–507. 15486304 10.1126/science.1100907

[bib51] Maoz U, Rutishauser U, Kim S, Cai X, Lee D, Koch C. Predeliberation activity in prefrontal cortex and striatum and the prediction of subsequent value judgment. Front Neurosci 2013; 7: 225. 24324396 10.3389/fnins.2013.00225 PMC3840801

[bib52] Grimm O, Heinz A, Walter H, Kirsch P, Erk S, Haddad L et al. Striatal response to reward anticipation: evidence for a systems-level intermediate phenotype for schizophrenia. JAMA Psychiatry 2014; 71: 531–539. 24622944 10.1001/jamapsychiatry.2014.9

[bib53] Szeszko PR, Robinson DG, Ikuta T, Peters BD, Gallego Ja, Kane J et al. White matter changes associated with antipsychotic treatment in first-episode psychosis. Neuropsychopharmacology 2014; 39: 1324–1331. 24549105 10.1038/npp.2013.288 PMC3988536

[bib54] Haijma SV, Van Haren N, Cahn W, Koolschijn PCMP, Hulshoff Pol HE, Kahn RS. Brain volumes in schizophrenia: a meta-analysis in over 18 000 subjects. Schizophr Bull 2013; 39: 1129–1138. 23042112 10.1093/schbul/sbs118 PMC3756785

[bib55] Bohlken MM, Mandl RCW, Brouwer RM, van den Heuvel MP, Hedman AM, Kahn RS et al. Heritability of structural brain network topology: a DTI study of 156 twins. Hum Brain Mapp 2014; 35: 5295–5305. 24845163 10.1002/hbm.22550 PMC6868932

