# Supplementary material for: Reduced fronto–striatal white matter integrity in schizophrenia patients and unaffected siblings: a DTI study
Source: NPJ Schizophr. 2015 Apr 1;1:15001–. doi: 10.1038/npjschz.2015.1 (PMC4849442; doi:10.1038/npjschz.2015.1)
Supplement: Supplementary Information [file npjschz20151-s1.doc]

**Table S1.** Antipsychotic medication in the patient group.

| **Medication** | **Frequency** | **Doses (range)** |
| --- | --- | --- |
| Clozapine | 7 | 200 – 600 mg d.d. |
| Olanzapine | 6 | 5 – 30 mg d.d. |
| Risperidon | 4 | 2 – 4 mg d.d. |
| Aripiprazol | 3 | 15 – 20 mg d.d. |
| Quetiapine | 2 | 275 – 400 mg d.d. |
| Penfluridol | 1 | 4 mg d.d. |

Abbreviations: d.d., daily dose.

**Table S2.** Other psychotropic medication in the patient group.

| **Medication** | **Frequency** | **Doses (range)** |
| --- | --- | --- |
| Paroxetine | 4 | 20 – 50 mg d.d. |
| Citalopram | 4 | 20 – 40 mg d.d. |
| Clonazepam | 3 | 0.5 – 3 mg d.d. |
| Oxazepam | 1 | 10 mg d.d. |
| Temazepam | 1 | 20 mg d.d. |
| Alprazolam | 1 | 1 mg d.d. |
| Lithium | 1 | 1000 mg d.d. |
| Biperiden | 1 | 2 mg d.d. |

Abbreviations: d.d., daily dose.
